# Modelling targeted rabies vaccination strategies for a domestic dog population with heterogeneous roaming patterns

**DOI:** 10.1371/journal.pntd.0007582

**Published:** 2019-07-08

**Authors:** Emily G. Hudson, Victoria J. Brookes, Salome Dürr, Michael P. Ward

**Affiliations:** 1 Sydney School of Veterinary Science, The University of Sydney, Camden, Australia; 2 Veterinary Public Health Institute, University of Bern, Liebefeld, Switzerland; 3 Charles Sturt University, School of Animal and Veterinary Sciences, Faculty of Science, Wagga Wagga, NSW 2678, Australia; Universidad Nacional Mayor de San Marcos, PERU

## Abstract

Australia is currently canine rabies free. However, communities located on the northern coastline–such as the Northern Peninsula Area (NPA), Queensland–are at risk of an incursion due to their large populations of susceptible free-roaming dogs and proximity to rabies-infected Indonesian islands. A rabies-spread model was used to simulate potential outbreaks and evaluate various disease control strategies. A heterogeneous contact structure previously described in the population of interest–*explorer* dogs, *roamer* dogs and *stay-at-home* dogs–was incorporated into the model using six spatial kernels describing contacts between dog roaming categories. Twenty-seven vaccination strategies were investigated based on a complete block design of 50%, 70% and 90% coverage for each of the three roaming categories to simulate various targeted vaccination strategies. The 27 strategies were implemented in four population structures in which the proportion of dogs in each category varied–*explorer* dominant, *roamer* dominant, *stay-at-home* dominant and a field population (based on field estimates of population structure). The overall vaccination coverage varied depending on the subpopulation targeted for vaccination and the population structure modelled. A total of 108 scenarios were simulated 2000 times and the model outputs (outbreak size and duration) were compared to Strategy 14 (a standard recommended overall 70% vaccination coverage). In general, targeting *explorer* dogs–and to a lesser extent *roamer* dogs–produced similar outbreaks to Strategy 14 but with a lower overall vaccination coverage. Similarly, strategies that targeted *stay-at-home* dogs required a higher vaccination coverage to produce significantly smaller and shorter outbreaks. This study provides some theoretical evidence that targeting subpopulations of dogs for vaccination based on their roaming behaviours (and therefore risk of rabies transmission) could be more efficient than blanket 70% vaccination campaigns. Such information can be used in preparedness planning to help improve control of a potential rabies incursion in Australia.

## Author summary

Australia is currently free of canine rabies. However, northern communities are at risk of an incursion. The free-roaming dogs in the at-risk communities of the Northern Peninsula Area, Queensland, Australia, display various roaming behaviours which affect how they interact with each other and subsequently affects how rabies could be spread within the population. The presence of these different roaming behaviours provides an opportunity to assess targeted vaccination strategies. Using a rabies spread model, this study provides evidence that targeting roaming dogs (that cause rapid rabies spread) for vaccination instead of dogs that remain around their owner’s residence, can be more efficient than the recommended overall 70% vaccination coverage to control rabies. Furthermore, if dogs that stay at home most of the time are targeted versus other roaming dogs, a higher overall coverage and more resources would be required to control a potential rabies outbreak than the overall 70% vaccination coverage. Comparing various vaccination strategies to find efficient and effective response options is beneficial to contribute to emergency planning and increase preparedness to control a rabies outbreak, should it occur.

## Introduction

Rabies is a viral encephalitis, estimated to cause approximately 59,000 human deaths worldwide; of these deaths, 99% are caused by dog bites [1]. Currently, the rabies-free communities of the Northern Peninsula Area (NPA), Queensland, Australia are at risk of a rabies incursion due to proximity to rabies-infected islands of Indonesia [2]. These communities also have large populations of free-roaming domestic dogs that are capable of transmitting and maintaining the rabies virus [3–4]. Simulation models can be used to assess different control strategies and inform decision makers on best practice if an outbreak was to occur, to improve preparedness and reduce the risk of disease spread.

Models that simulate direct-contact disease transmission benefit from incorporating movement and contact patterns of the at-risk population. In the NPA, three roaming patterns in the domestic dog population have been recently described–*explorer* dogs who frequently roam away from their owner’s residence, *roamer* dogs who mainly remain around their owner’s residence but roam away sometimes, and *stay-at-home* dogs who spend most (if not all) of their time around their owner’s residence, roaming to only the nearest neighbours [5]. These roaming patterns have been shown to create heterogeneous contact probabilities between individuals in the population and have been described by six spatial contact kernels based on all possible combinations of categories between a pair of dogs [6]; two *explorer* dogs (EE kernel), an *explorer* dog and a *roamer* dog (ER kernel), an *explorer* dog and a *stay-at-home* dog (ES kernel), two *roamer* dogs (RR kernel), a *stay-at-home* dog and a *roamer* dog (SR kernel) and two *stay-at-home* dogs (SS kernel). Previously, these kernels were individually incorporated into a rabies-spread model for the NPA developed by Dürr and Ward [3]. This was to study in isolation the differences in the contact probabilities produced by the spatial kernels and to quantify the effects of the different contact probabilities on predicted disease spread [3, 6]. However, heterogeneous contacts within the population can greatly affect epidemic spread and subsequent model predictions [7]. Therefore, to model the spread of rabies through the NPA dog population more accurately, all six kernels need to be used in the NPA rabies-spread model to describe the heterogeneous contact structure in this population.

The representation of a heterogeneous contact structure in the NPA rabies-spread model not only leads to more probable outbreak scenarios and predictions, it also allows exploration of targeted vaccination strategies for specific dog sub-populations in this area. Vaccination is the most effective control strategy to limit the spread of rabies in the reservoir population and exposure to humans, and 70% vaccination coverage of dogs has been recommended to eliminate rabies [8–9]. Several countries–including Tanzania [10], Indonesia [11], Chad [12–13] and several in Latin America [14]–have experienced a reduction in rabies transmission and human cases by reaching a 70% vaccination coverage in the dog population. Modelling has also shown that annual dog vaccination that reaches 70% coverage is sufficient for rabies elimination [15–17]. With limited resources, vaccination programs should be tailored to suit the region’s individual characteristics–including human-dog interaction and dog population ecology–to efficiently and cost-effectively reduce rabies spread. For example, Kaare et al. [18] found that centre point vaccination (a common approach in rabies endemic regions in which vaccination stations are set up and owners bring dogs to the station) alone reached a 70% coverage and was cost-effective in agro-pastoralist communities in Tanzania. However, centre point vaccination alone was insufficient to reach 70% coverage and more expensive in remote pastoralist communities of Tanzania [18]. The three roaming categories of dogs in the NPA described by Hudson et al. [6], provides an opportunity to use the NPA rabies model developed by Dürr and Ward [3] to evaluate targeted vaccination strategies. Targeting *explorer* and *roamer* dogs for vaccination in preference to *stay-at-home* dogs could produce the same or better results as the recommended overall 70% vaccination coverage potentially with fewer resources such as vaccine numbers. This is because *explorer* and *roamer* dogs travel longer distances and subsequently come into contact with more dogs, producing a higher probability of transmitting rabies compared to *stay-at-home* dogs [6].

Previous rabies control modelling studies have explored targeted vaccination strategies in which high-risk populations are prioritised over lower risk populations. For example, a previous study modelled canine rabies spread in the dog populations of the Serengeti District, Tanzania as a metapopulation model with various sub-populations [19]. The study evaluated vaccination strategies that either targeted sub-populations that would result in the greatest decrease in global risk (disease occurrence), or targeted sub-populations based only on their relative size, and assessed their performance based on their reduction of mean rabies occurrence compared to an unvaccinated population. The former strategy was found to be up to 62% more effective at reducing mean rabies occurrence compared to the latter strategy. Another study that used a network model to investigate rabies spread in Chad found vaccination strategies that either targeted dogs with higher degree centrality, betweenness centrality, or dogs that roam larger areas, reduced outbreak size and probability more than a random vaccination strategy [20]. The above modelling studies provide theoretical evidence that targeting high-risk populations for vaccination is effective.

The objectives of this study were to incorporate the six spatial contact kernels for each of the roaming categories and their combinations created by Hudson et al. [6] into the rabies-spread model for the NPA developed by Dürr and Ward [3], to more accurately represent the heterogeneous contact structure of the dog population. With this updated model, we then explore reactive vaccination strategies that target the individual dog categories within the NPA population at different coverage levels, and compare model predictions to the recommended 70% population vaccination coverage. The results could then be used by decision makers to further investigate and develop reactive vaccination programs in the event of a rabies outbreak in the NPA.

## Methods

### Study site

The NPA is a local government area located at the tip of Cape York Peninsula, Queensland, Australia. It consists of five Aboriginal and Torres Strait Islander communities–Seisia, New Mapoon, Bamaga, Umagico and Injinoo. Most of the human population (99%; N = 2773) live within these communities [21]. The total dog population in the five NPA communities is estimated to be 813 (range = 770–8680) and most of these dogs are free-roaming [4]. It is believed that most if not all dogs in these communities are owned [4].

### Simulation model

The rabies-spread simulation model used was previously developed by Dürr and Ward [3] and modified by Hudson et al. [6]. Model parameters are shown in S1 Table. Briefly, the modifications included updating the dog population size (813 dogs) and spatial arrangement of dog-owning houses in the communities–based on recent demographic studies of the NPA dog population [4]–and the inclusion of two parameters to describe the probability of a clinically infected dog developing furious rabies and a subsequent increased bite probability of a furious rabid dog. In addition, the model was modified in the current study to include a birth and death rate to simulate a dynamic steady-state population. Based on data collected by Hudson et al. [4], 12.6% of the adult dog population (1 year and older) die each year. The model runs on a daily time step and so the death rate was converted to a daily probability of death and a Poisson distribution was used to determine how many dogs are randomly selected to die each day from cause other than rabies. Any live dog in the model can be randomly selected to die, regardless of rabies infection status. The birth rate used in the model was chosen to be equivalent to the death rate to simulate a steady-state population. Again, a Poisson distribution was used to determine how many new dogs are added to the population at the beginning of each day. Dog-owning houses were selected at random to receive the new dogs. The random selection of houses was with replacement, so that one house can receive multiple new dogs.

### Kernel incorporation and population structures

The six spatial kernels developed by Hudson et al. [6] describe the daily probability of contact between pairs of dogs dependent on the distance between their residences and the roaming behaviour of the dogs (*explorer*, *roamer* or *stay-at-home* type of dog). All dogs in the population are assumed to be susceptible and are in contact with other dogs, regardless of roaming category. These kernels were used simultaneously in the model. For example, if the infected dog is an *explorer* (E) dog and the contacted dog is a *roamer* (R) dog, the ER kernel is used to estimate their daily probability of contact. If both dogs are a *stay-at-home* (S) dog then the SS kernel is used to estimate the daily probability of contact. The current proportions of dogs in each roaming category are unknown in the NPA. Therefore, three population structures were modelled and compared–Explorer Dominant (ED) in which 60%, 20% and 20% of the population are *explorer* dogs, *roamer* dogs and *stay-at-home* dogs, respectively, Roamer Dominant (RD) in which 20%, 60% and 20% of the population are *explorer* dogs, *roamer* dogs and *stay-at-home* dogs, respectively, and Stay-at-home Dominant (SD) in which 20%, 20% and 60% of the population are *explorer* dogs, *roamer* dogs and *stay-at-home* dogs, respectively. A fourth population structure based on roaming category proportions of dogs collected from field data (9 *stay-at-home* dogs and 6 *roamer* and *explorer* dogs each) in Hudson et al. [5] was also modelled (Field Population; FP) in which 29%, 29% and 42% of the population are *explorer* dogs, *roamer* dogs and *stay-at-home* dogs, respectively. All dogs in the model were randomly assigned a roaming category at the beginning of each simulation based on the respective population structure ratios, and the chosen roaming category for each dog was retained until the end of the simulation. New dogs added to the population from the birth rate function were also randomly assigned a roaming category based on these ratios. For example, the probability a new dog will be an *explorer* dog in the ED population is 60% but only 20% in the RD and SD populations and 29% in the FP population.

### Vaccination strategies

Full details of the rabies simulation model and how reactive vaccination strategies are implemented are described in Dürr and Ward [3]. Briefly, a vaccination campaign is triggered after detection of the first case with clinical signs and the delay (7 days) to acquire vaccines and organise the campaign, which is simulated as a door-to-door campaign. All dogs in the population that are not yet clinically rabid or vaccinated are available for vaccination. The number of dogs selected as vaccination candidates is dependent on the vaccination capacity (default 50 dogs/day based on semi-structured interviews with people with local knowledge and knowledge of Australia’s biosecurity responses) and the overall target population vaccination coverage. For example, if the desired overall vaccination coverage is 70% and the vaccination capacity is 50 dogs/day, the 72 dogs with the closest residences to all detected cases are selected as vaccination candidates that day. From these candidates, 70% (which is the vaccination capacity of 50 dogs) are randomly chosen to be vaccinated, subsequently producing 70% vaccination coverage. The vaccination campaign is continued each day until the aimed coverage (e.g. 70%) of the entire dog population was reached. Vaccination coverages of 90%, 70% and 50% were trialled for each roaming category in a complete block design to produce 27 vaccination strategies for each population structure (Table 1). The overall population-level vaccination coverages varied dependent on the population structured used. The selected vaccination candidates were grouped into their roaming categories and the individual category vaccination coverages were applied to randomly select dogs for vaccination within their respective category. For example, if the vaccination coverage for the *stay-at-home*, *roamer* and *explorer* categories were 70%, 90% and 50% respectively, then 70%, 90% and 50% of the *stay-at-home*, *roamer* and *explorer* dogs chosen as vaccination candidates were finally vaccinated, respectively.

**Table 1:** Vaccination strategies and the overall population coverage–based on various vaccination coverages for each roaming category in the dog population–tested in a rabies-spread simulation model on four dog population structures in the Northern Peninsula Area, Queensland, Australia. Each strategy was simulated 2000 times.

Strategy	Vaccination coverage of *explorer* dogs (%)	Vaccination coverage of *roamer* dogs (%)	Vaccination coverage of *stay-home* dogs (%)	Overall vaccination coverage for ED (%)	Overall vaccination coverage for RD (%)	Overall vaccination coverage for FP (%)	Overall vaccination coverage for SD (%)
1	50	50	50	50	50	50	50
2	50	50	70	54	54	58	62
3	50	50	90	58	58	67	74
4	50	70	50	54	62	56	54
5	50	70	70	58	66	64	66
6	50	70	90	62	70	73	78
7	50	90	50	58	74	62	58
8	50	90	70	62	78	70	70
9	50	90	90	66	82	78	82
10	70	50	50	62	54	56	54
11	70	50	70	66	58	64	66
12	70	50	90	70	62	73	78
13	70	70	50	66	66	62	58
14	70	70	70	70	70	70	70
15	70	70	90	74	74	78	82
16	70	90	50	70	78	67	62
17	70	90	70	74	82	76	74
18	70	90	90	78	86	84	86
19	90	50	50	74	58	62	58
20	90	50	70	78	62	70	70
21	90	50	90	82	66	78	82
22	90	70	50	78	70	67	62
23	90	70	70	82	74	76	74
24	90	70	90	86	78	84	86
25	90	90	50	82	82	73	66
26	90	90	70	86	86	82	78
27	90	90	90	90	90	90	90

The 27 vaccination strategies were modelled for the four population structures to produce 108 scenarios. Each scenario was simulated 2000 times, which is sufficient for convergence of summary statistics of outbreak outputs for this simulation model [6]. To better compare the effect of vaccination on the outbreak size (number of rabid dogs) and outbreak duration (days), simulations within each vaccination strategy in which vaccination was not triggered (either because of no propagation of rabies or disease fade-out before vaccination was triggered) were removed after the original 2000 simulations were complete. Model simulations were conducted on The University of Sydney’s High Performance Computer using the R statistical program [22].

### Statistical analysis

Within each population structure, a Kruskal-Wallis test was performed to determine overall significance of differences in outbreak size and duration between the 27 vaccination strategies. A Dunn’s Test with Bonferroni correction was used as a post-hoc test to compare the vaccination strategies to Strategy 14 (which represented 70% vaccination coverage in all three roaming categories) in a pairwise fashion at a 0.01 significance level. Statistical analyses were performed in R [22].

## Results

**Fig 1:**
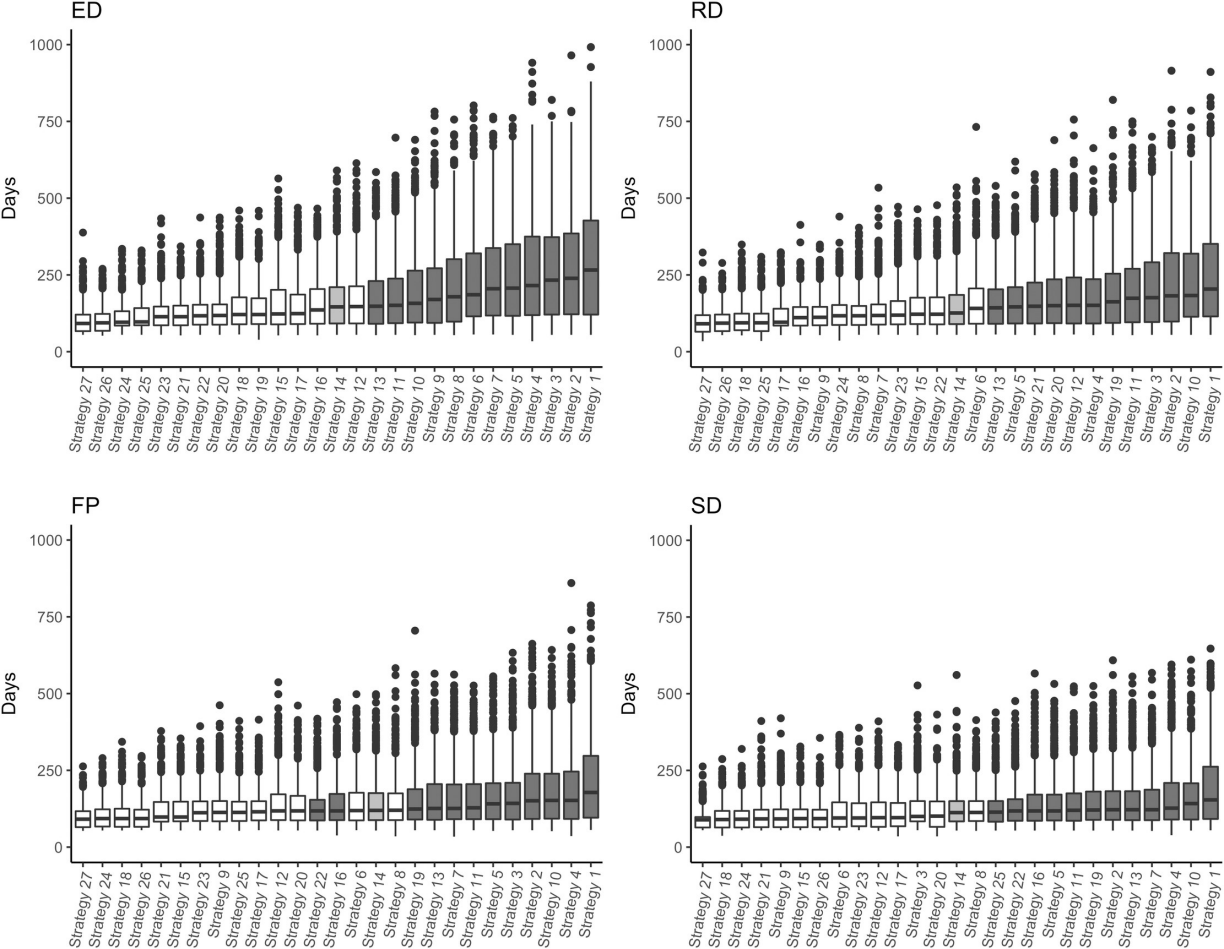
Boxplots of simulated rabies outbreak durations (days) from 27 vaccination strategy scenarios predicted by a rabies-spread model for the Northern Peninsula Area, Queensland, Australia in four population structures–ED, *Explorer* Dominant; RD, *Roamer* Dominant; SD, *Stay-at-home* Dominant; FP, Field Population. The strategy that represents a randomly distributed 70% overall vaccination coverage (Strategy 14) is shaded light grey. The strategies that had an overall vaccination coverage <70% are highlighted in dark grey. The boxplots are ordered in ascending order of median outbreak duration.

**Fig 2:**
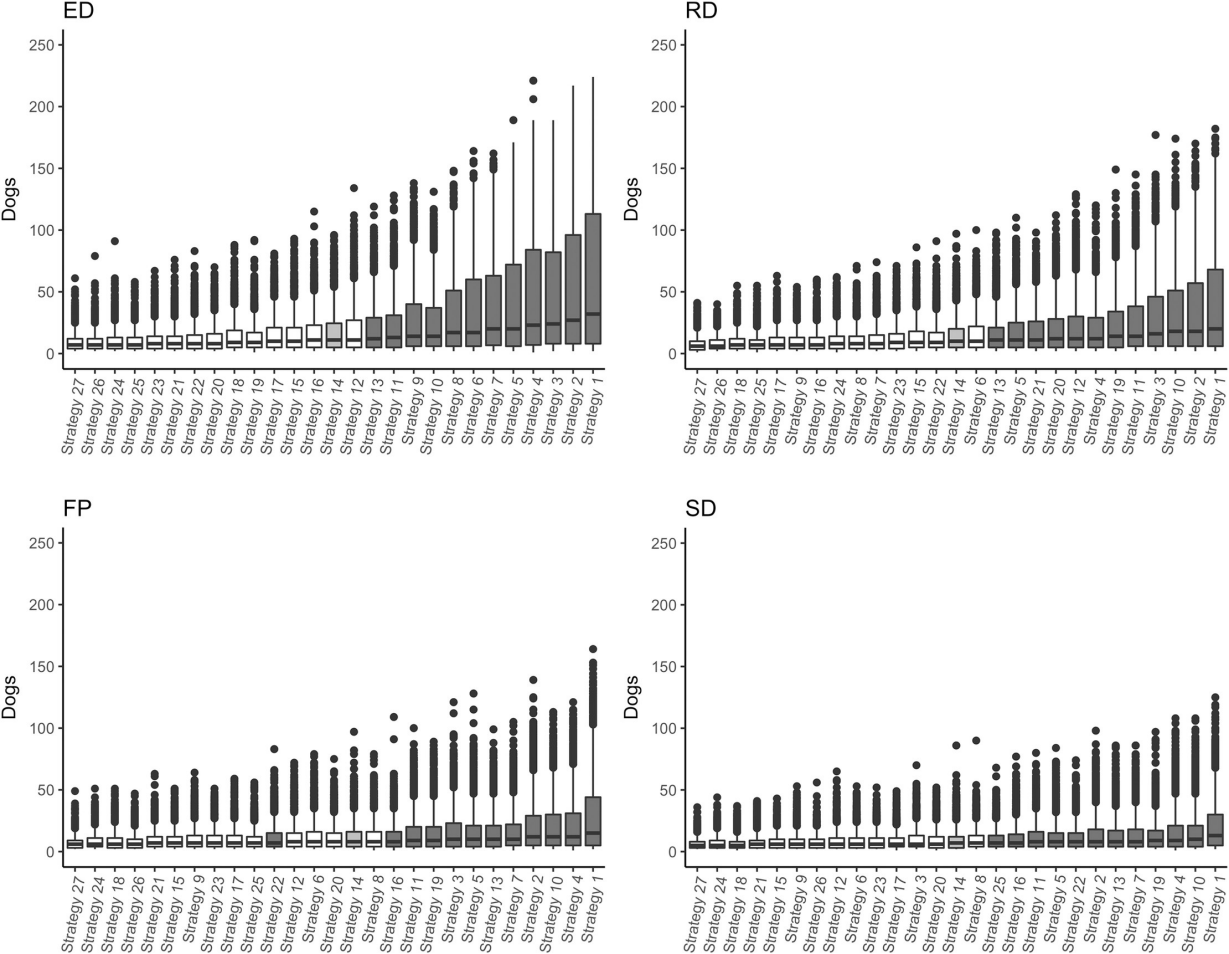
Boxplots of simulated number of rabid dogs from 27 vaccination strategy scenarios predicted by a rabies spread model for the Northern Peninsula Area, Queensland, Australia four population structures–ED, *Explorer* Dominant; RD, *Roamer* Dominant; SD, *Stay-at-home* Dominant; FP, Field Population. The strategy that represents a randomly distributed 70% overall vaccination coverage (Scenario 14) is shaded grey. The strategies that had an overall vaccination coverage <70% are highlighted in dark grey. The boxplots are ordered in ascending order of median outbreak size.

From all 216,000 simulations, vaccination was triggered in 184,968 (86%). The ED population had the highest percentage of simulations in which vaccination was triggered (88%) followed by the RD population (87%), whereas the FP and SD had the smallest (85% and 83%, respectively). Only simulations in which vaccination was triggered were further analysed. For all population structures, Strategy 27 (vaccination of all roaming categories at 90%) was the most effective strategy to control rabies with the shortest outbreak duration (days; Fig 1) and smallest outbreak size (number of rabid dogs; Fig 2). For all strategies mentioned below, the vaccination coverages of the three roaming categories are reported in brackets immediately after the strategy number in the order of *explorer*, *roamer* and *stay-at-home*. For example, Strategy 22 (90–70–50) represents Strategy 22 that vaccinated *explorer* dogs at 90%, *roamer* dogs at 70% and *stay-at-home* dogs at 50%.

### Stay-at-home Dominant (SD) population structure and Field Population (FP) structure

The results for these population structures are presented together because their results were similar. Outbreaks tended to be smaller with shorter durations and less variation compared to the RD and ED population structures (Fig 1 and Fig 2). In the SD population structure, Strategy 16 (70–90–50), Strategy 22 (90–70–50) and Strategy 2 (50–50–70) had the same overall vaccination coverage (62%), lower than the overall 70% vaccination coverage in all dog categories (non-targeted vaccination) of Strategy 14 (70–70–70). However, only Strategies 16 and 22, which focussed on vaccinating *explorer* and *roamer* dogs, produced similar outbreaks to Strategy 14. Strategy 2 focussed on vaccinating *stay-at-home* dogs and produced significantly larger and longer outbreaks than Strategy 14. Also, Strategy 25 (90–90–50) which had an overall vaccination coverage of 66% produced similar outbreaks to Strategy 14, unlike Strategy 5 (50–70–70) and Strategy 11 (70–50–70), which has the same overall vaccination coverage of 66% but produced significantly different outbreaks than Strategy 14. Furthermore, Strategy 6 (50–70–90), Strategy 12 (70–50–90) and Strategy 26 (90–90–70) had an overall population coverage of 78% (higher than Strategy 14), but only Strategy 26, which focussed on vaccinating *explorer* and *roamer* dogs, performed significantly better than Strategy 14.

Similar to the SD population, Strategy 16 (70–90–50) and Strategy 22 (90–70–50)–both with overall vaccination coverage of 67%–produced similar outbreaks to Strategy 14 (70–70–70) for the FP population structure. Strategy 3 (50–50–90)–which has the same overall vaccination coverage (67%) as Strategies 16 and 22 –focussed on vaccinating *stay-at-home* dogs and produced significantly larger and longer outbreaks than Strategy 14. Strategy 6 (50–70–90), Strategy 12 (70–50–90) and Strategy 25 (90–90–50) all have an overall vaccination coverage of 73%. However, only Strategy 25 –which targets *explorer* and *roamer* dogs over *stay-at-home* dogs–produced significantly smaller and shorter outbreaks than Strategy 14. Similarly, Strategy 9 (50–90–90)–which focussed on vaccinating *roamer* and *stay-at-home* dogs over *explorer* dogs–produced similar sized outbreaks compared to Strategy 14, even with a higher overall vaccination coverage of 78%.

### Explorer Dominant (ED) population structure

The ED population tended to produce the largest and longest outbreaks compared to the other population structures, especially when overall vaccination coverage was low (Fig 1 and Fig 2). Strategy 11 (70–50–70) and Strategy 13 (70–70–50) produced similar outbreaks to Strategy 14 (70–70–70) with a lower overall vaccination coverage of 66%. Conversely, Strategy 9 (50–90–90)–which also has an overall vaccination coverage of 66%–produced significantly larger and longer outbreaks to Strategy 14. Furthermore, of Strategies 15 (70–70–90), 17 (70–90–70) and 19 (90–50–50) with an overall vaccination coverage of 74% each, only Strategy 19 –which focused on vaccinating *explorer* dogs over the other categories–performed better than Strategy 14.

### Roamer Dominant (RD) population structure

The RD population generally produced larger and longer outbreaks than the FP and SD populations but smaller and shorter outbreaks than the ED population (Fig 1 and Fig 2). Strategy 5 (50–70–70), Strategy 13 (70–70–50) and Strategy 21 (90–50–90; overall vaccination coverage of 66% each) produced similar sized outbreaks to Strategy 14 (70–70–70). Strategies 5 and 13 also produced similar outbreak durations, whereas Strategy 21 produced significantly longer durations. Strategy 7 (50–90–50), Strategy 15 (70–70–90) and Strategy 23 (90–70–70) had a higher overall vaccination coverage (74%) than Strategy 14. However, only Strategy 7 performed significantly better than Strategy 14 for both outbreak size and duration. Strategy 23 produced significantly shorter outbreaks than Strategy 14, but no significant difference in outbreak size was found.

## Discussion

The most effective vaccination strategy (smallest and shortest outbreaks) assessed in this study for all population structures was Strategy 27 (90–90–90% of all subpopulations). However, to achieve this coverage during an outbreak would require a considerable amount of resources and labour, likely limiting in a remote area such as the NPA. Also, 70% population coverage has been shown to effectively limit rabies spread and potentially lead to elimination [8–9]. Therefore, a 90% population coverage may not be necessary. Although the 70% coverage recommendation for halting rabies transmission in Coleman and Dye [8] is based on epidemic outbreak data from the United States [23], Malaysia [24], Indonesia [25] and Mexico [26], it has been mainly used and assessed in rabies endemic regions. This level of coverage has also been successful in limiting spread following the 2008 Bali rabies outbreak [11]. Given the adoption of 70% vaccination coverage as a recommended standard for rabies control, it was used in the present study to compare the effectiveness of alternative strategies.

This study provides theoretical evidence that targeting subpopulations of dogs at higher risk–*explorer* or *roamer* dogs–instead of *stay-at-home* dogs could be more resource efficient than the recommended 70% coverage (i.e. no targeting of subpopulations; Strategy 14). The efficiency of a vaccination strategy to perform similarly to Strategy 14 (70–70–70) with fewer resources depended on whether it was *explorer* dogs, *roamer* dogs, or both categories that were targeted for higher vaccination coverage rather than *stay-at-home* dogs. For example, in the FP and SD population structures, targeting both *explorer* and *roamer* dogs at either 70% or 90%–regardless of the *stay-at-home* coverage–gave similar outbreaks to Strategy 14 with a lower overall vaccination coverage. In the ED population *explorer* dogs had to be targeted at 70% coverage and either *roamer* or *stay-at-home* dogs also at 70% coverage for the strategy to be similar to Strategy 14 with fewer resources. Finally, in the RD population, *roamer* dogs had to be targeted with 70% coverage and either *explorer* or *stay-at-home* dogs also with 70% coverage for the strategy to be similar to Strategy 14. These findings are similar to those of Laager et al. [20] in which targeted vaccination strategies for high-risk dogs (high betweenness centrality, large area roamed, and high degree centrality) were more effective at reducing outbreak probability and outbreak size compared to a random vaccination strategy when modelled in a network simulation of rabies spread in Chad [20].

The success of targeting *explorer* and *roamer* dogs versus *stay-at-home* dogs in the SD and FP population structures suggests that it is more efficient to target high-risk dogs rather than the most abundant type of dogs. This is consistent with output from another rabies model developed for Tanzania, which compared vaccination strategies that either prioritised the largest subpopulations for vaccination or subpopulations that would cause the largest reduction in global risk [19]. For the high-risk vaccination strategies, sub-populations had risk scores defined by multiple factors, including probability of rabies occurrence in each sub-population, spatial arrangement of sub-populations, distance to other possible sources of infection and population size. Conversely, only population size was a factor for population prioritisation of vaccination. In this study, it was found that the high-risk prioritised vaccination strategies reduced mean rabies occurrence by 33.4% from an unvaccinated population compared to the population size prioritised strategy, which reduced mean rabies occurrence by 16.9%. In the risk-prioritised strategy, the largest subpopulation received no vaccine because it would cause the smallest reduction of global risk compared to other high-risk, smaller subpopulations [19]. The results in the current study, in conjunction with previous modelling studies, provide theoretical evidence that targeting high-risk dogs (*explorer* and *roamer* dogs) is likely to be a more resource efficient approach in the event of a rabies outbreak in the NPA. The results can therefore be used by decision makers to develop a reactive vaccination policy by providing justification for further investigations into operational logistics to practically implement these findings, or direct investigations on larger dog populations in other geographical regions to test if these findings can be generalised.

Previous studies have suggested that far-roaming dogs (*explorer* dogs in our study) are important for disease spread, including rabies [27–29]. Hudson et al. [6] demonstrated that rabies infection in *explorer* dogs can result in fast developing and large outbreaks, but suggested that “mid-roaming” (*roamer*) dogs are also important for rabies spread. *Explorer* dogs roam away from their owner’s residence frequently whereas *roamer* dogs mostly remain around their owner’s residence, only roaming away occasionally. Further to Hudson et al. [6], this study also demonstrates the importance of *roamer* dogs. For example, in the RD population, Strategy 7 (50–90–50)–which has an overall vaccination coverage of 74%–performs significantly better than Strategy 14 but Strategy 23 (90–70–70)–which also has an overall vaccination coverage of 74%–only reduces outbreak duration but not size. This demonstrates targeting *roamer* dogs for vaccination was more important than targeting *explorer* dogs when comparing strategies with similar vaccination coverages. This differs from the other population structures modelled in which the level of *explorer* dog, and sometimes *roamer* dog, vaccination was important when comparing strategies with similar overall population vaccination coverage. Also, not only do *roamer* dogs influence spread within the dog population, they are likely to have more human contact than *explorer* dogs due to the relative longer time spent at home [5], and could be an important source of human infection. Therefore, targeted vaccination of *explorer* and *roamer* dogs could be important to reduce the risk of transmission in both the dog and human population during an outbreak.

In the current response plan for a rabies outbreak in Australia (AUSVETPLAN), there are no specific strategies for implementing a vaccination campaign, rather the goal should be to vaccinate as many dogs as possible [30]. This could make Australia vulnerable to an increased risk of delayed rabies control. For example, centre point vaccination stations and blanket house-to-house vaccination strategies are commonly used in rabies infected areas [10, 11, 18, 31]. If these general strategies were implemented as a response to a rabies outbreak in the NPA (our study area), *stay-at-home* dogs would more likely be presented at vaccination points or vaccinated at their residence because they are more accessible and available (compared to *explorer* and *roamer* dogs) [5]. According to the results in this study, inadvertently targeting *stay-at-home* dogs versus *explorer* and *roamer* dogs–e.g. Strategy 6 (70–50–90) and Strategy 12 (70–50–90)–would require a higher overall vaccination coverage than 70%, and subsequently more resources, to be as effective as Strategy 14 (70–70–70). For example, Strategy 12 (70–50–90) in the SD population had a higher overall vaccination coverage than Strategy 14 (78% and 70%, respectively), but produced similar outbreaks to Strategy 14, and therefore was not more effective. Furthermore, a lack of acceptable means of identifying vaccinated animals could exacerbate the problem [30]. Without identification of vaccinated dogs, resources could be wasted revaccinating or culling vaccinated dogs [11]. Common identification methods include collars or microchips. However, microchipping would be laborious and time consuming and collars are rarely used and are known to be impractical in the NPA (a high percentage of collar loss has been reported when these were used for GPS studies [5]). Therefore, preparedness against rabies in the NPA can be improved by considering targeted vaccination strategies in emergency planning as well as by investigating effective and practical vaccination identification methods.

The population structure in terms of roaming category proportions in the NPA is unknown. Due to the general success of strategies that target *explorer* and *roamer* dogs versus *stay-at-home* dogs across the various population structures modelled in this study, the evidence suggests that in the case of an unknown population structure, targeting *explorer* and *roamer* dogs would increase the success of a vaccination campaign to control the spread of rabies with limited resources. However, it is difficult to determine the roaming category of dogs without GPS data collection and analysis. There are a few studies that investigated the influences and predictors (such as sex and reproductive status) of roaming for free-roaming domestic dogs [28, 29, 32]. This research needs to be expanded to include the predictors of roaming without reliance on extensive GPS studies so that vaccination campaigns can be better targeted. However, the practical recommendations from this study is to vaccinate dogs that are currently roaming in the NPA, which would, by definition, target the *roamer* and *explorer* dogs instead of *stay-at-home* dogs. Therefore, detailed knowledge of individual dogs’ roaming category might not be required for effective vaccination targeting. A potential vaccination method could be vaccination baits in strategic areas which would indirectly target roaming dogs (*explorer* and *roamer* dogs) instead of *stay-at-home* dogs, without prior knowledge of roaming category, which has also been suggested by Laager et al. [20]. Some studies have shown oral vaccinations to be an effective complementary method to parenteral vaccinations in dog populations that are free-roaming [33–36], and could be considered when developing vaccination campaigns for the free-roaming dog populations of the NPA. Cost efficiency was not considered in this analysis because the main aim was to explore resource efficient vaccination strategies in an area which is resource poor and provide some information on resource allocation that still effectively reduces rabies spread. The theoretical results in this study–targeting *explorer* or *roamer* dogs versus *stay-at-home* dogs–can be used to justify and direct future studies such as a cost-benefit analysis to help develop implementation of cost-efficient targeted reactive vaccination campaigns. For example, a recent study in India found that oral bait hand-outs vaccinated 35 dogs/person/day and was more cost-efficient when compared to a capture-vaccinate-release method (9 dog/person/day) for vaccinating roaming dogs that were difficult to handle or not at the owner’s residence [37].

The dog roaming categories were randomly assigned in the model allowing for multiple roaming categories per house. Roaming category is likely clustered by house–there is likely a higher probability of dogs in the same house to have the same roaming behaviours. There is some evidence that spatial clustering within a population could influence a rabies outbreak and vaccination campaigns [19, 38]. However, the dog populations in these studies are modelled as metapopulations with spatially separate sub-populations, rather than dogs relatively spatially homogenous but clustered by individual traits like roaming behaviour. The current model is spatially explicit for individual dogs. Therefore, further studies should investigate the effects of clusters of dogs with the same roaming category on the predicted spread of rabies. Alternatively, a network model might be more suitable to capture the complexities of the contact structure and roaming category distribution because it can account for individualised contacts and pack roaming, unlike the current model. A social network model has been developed for three islands in the Torres Strait [39]. However, the information and resources needed to create a social network model in the NPA would be vast due to the large dog population (approximately 813 dogs) [4] compared to 40–45 dogs on these small islands [40].

The results in this study apply to a steady-state dynamic population. The birth and death rate used in this current study were modest (median number of dogs born and naturally died in all outbreaks = 40). However, in dog populations with high turnover or high growth populations herd immunity wanes because of influxes of susceptible dogs which can hinder elimination campaigns [15, 17, 41]. Therefore, in a growing or high turnover population, the outbreaks will likely be larger and longer (only 4% of all outbreaks lasted longer than a year in this study) and the vaccination strategies might perform differently–overall vaccination coverage might not be achieved with targeted strategies, or targeting *explorer* and *roamer* dogs could be even more critical to stop rabies spread. Some studies have modelled vaccination campaigns in high turnover dog populations and have found that annual vaccination with 70% coverage is sufficient to maintain herd immunity above a critical level [42, 43]. However, these studies were performed on rabies endemic populations. Therefore, further research into the effects of targeted vaccination campaigns on a growing or high turnover population during an epidemic is needed.

This study provides promising theoretical evidence that targeting *explorer* and *roamer* subpopulations of dogs for vaccination campaigns instead of *stay-at-home* dogs could be more efficient than a random 70% overall vaccination coverage to control a rabies outbreak in the NPA. Practically, this means targeting dogs roaming during an outbreak (*explorer* and *roamer* dogs) for vaccination instead of more accessible or potentially more abundant non-roaming dogs (*stay-at-home* dogs) that stay around their owner’s residence. This information can be used to inform decision makers on potential resource efficient control measures in the event of a rabies incursion in the NPA, which can be used further to develop reactive vaccination strategies to improve Australia’s preparedness plans. How to efficiently target roaming dog subpopulations for vaccination in the field remains a challenge. There is a need for more research into novel vaccine delivery systems–for example, oral baits–that might allow roaming domestic dogs to be targeted to control rabies. This study and other modelling studies [19–20] can be used to direct further investigations into the usefulness of targeted vaccination in other regions to test the generalisation of the current findings.

## Supporting information

S1 Table Model parameters. Name, description, default values and source of all parameters used in the rabies simulation model adapted from Dürr and Ward (2015).
(DOCX)

S1 Zip file folder This folder contains 108 txt files for each model scenario. The files contain a summary for each simulation for its respective scenario. The files are named first with the population structure (FP, ED, RD, SD) and then the specific vaccination strategy used for that scenario.
(ZIP)
