# Supplementary material for: Modelling targeted rabies vaccination strategies for a domestic dog population with heterogeneous roaming patterns
Source: PLoS Negl Trop Dis. 2019 Jul 8;13(7):e0007582. doi: 10.1371/journal.pntd.0007582 (PMC6638970; doi:10.1371/journal.pntd.0007582)
Supplement: S1 Table — Name, description, default values and source of all parameters used in the rabies simulation model adapted from Dürr and Ward (2015). (DOCX) [file pntd.0007582.s001.docx]

**Model parameters.** Name, description, default values and source of all parameters used in the rabies simulation model adapted from [1].

| Parameter | description | Default value | Source |
| --- | --- | --- | --- |
| Max_time | how long (simulated days) the model should run | 10000 | ― |
| population parameters | | | |
| starting_population | dog population size at the start of the simulation | 813 | [2] |
| birth rate | assumed to be equal to death rate | 0.1260504 | - |
| death rate | proportion of population that dies per year - implemented as a Poisson distribution for daily number of dogs taken out of the population  for example, On the 1st day, Poisson is (813*0.1260504)/365 and a number from this distribution is selected as the number of dogs to be taken out of the population that day | 0.1260504 | [2] |
| Field population structure | proportion of dogs in each of the roaming dog categories for the Field population structure | Explorer category: 0.29,  Roamer category: 0.29,  Stay-at-home category: 0.42 | [3] |
| Stay-at-home Dominant structure | proportion of dogs in each of the roaming dog categories for the Stay-at-home Dominant structure | Explorer category: 0.2,  Roamer category: 0.2,  Stay-at-home category: 0.60 | assumption |
| Roamer Dominant structure | proportion of dogs in each of the roaming dog categories for the Roamer Dominant structure | Explorer category: 0.2,  Roamer category: 0.6,  Stay-at-home category: 0.2 | assumption |
| Explorer Dominant structure | proportion of dogs in each of the roaming dog categories for the Explorer Dominant structure | Explorer category: 0.4,  Roamer category: 0.2,  Stay-at-home category: 0.2 | assumption |
| index dog(s) related parameters | | | |
| Index_region | definition of the case(es) is hierarchical: if index_dog is defined, all other are not used in the model; else if index_community is defined, index_region is not used in the model | NA | ― |
| index_community |  | NA | ― |
| index_dog |  | NA | ― |
| nb_index_dogs |  | 1 | ― |
| disease states related parameters | | | |
| infectiousDelay | Incubation period: time period between exposure (bite) and infectiousness | Pert(22.8,25.8,29) | [4–6] |
| clinicalDelay | Subclinical period (time period between infectiousness and occurrence of typical rabies clinical signs; this parameter mainly influences the detection of rabies as the detection delay starts with the beginning of the clinical phase) | Unif(1,3) | [7,8] |
| mortalityDelay | Clinical period (time period between start of clinical signs and death) | Pert(2,4.7,12) | [4–6] |
| Prop_furious_dog | Proportion of dogs developing furious rabies | Unif(0.1,0.6) | [9] |
| Within and between household contact/transmission related arguments | | | |
| cont_prob_sameHH | Daily contact probability of dogs within the same household | Unif(0.94,1) | [1] |
| EE kernel | Distance kernels that define the probability of a daily contact for households located a given distance apart. The individual functions (Weibull, 3- or 4- parameter logistic) are used to create pert distributions  median = median,  minimum = 2.5%,  maximum = 97.5% | Median = Weibull (0.001303, -0.578108, -10.607419, 2.333159) | [10] |
|  |  | 2.5% = Weibull (0, -0.2095, -17.1428, 3.8934) | [10] |
|  |  | 97.5% = Logistic (0.9413, 0.004717, 113.7, 51.24) | [10] |
| ER kernel |  | Median = Weibull (0, -0.4352, -9.7898, 2.0414) | [10] |
|  |  | 2.5% = Weibull (0, -0.1929, -14.4917, 3.2238) | [10] |
|  |  | 97.5% = Logistic (0.75355, 0.01377, 158.81465, 53.71570) | [10] |
| ES kernel |  | Median = Weibull (0, -0.5189, -8.3546, 1.9440) | [10] |
|  |  | 2.5% = Logistic (0.1235, 60.3060, -10.0984) | [10] |
|  |  | 97.5% = Weibull (0.004483, -0.813974, -9.184410, 1.895362) | [10] |
| RR kernel |  | Median = Logistic (0.7863, 70.2314, -16.1642) | [10] |
|  |  | 2.5% = Weibull (0.000481, -0.577691, -11.069992, 2.745385) | [10] |
|  |  | 97.5% = Logistic (0.8598, 127.8663, -30.3622) | [10] |
| SR kernel |  | Median = Logistic (0.7776, 61.2093, -12.6910) | [10] |
|  |  | 2.5% = Logistic (0.5502, 36.8422, -12.9916) | [10] |
|  |  | 97.5% = Logistic (0.8591, 104.0302, -23.3257) | [10] |
| SS kernel |  | Median = Weibull (0, -0.8131, -12.2370, 2.9838) | [10] |
|  |  | 2.5% = Weibull (0, -0.5567, -11.2435, 2.9152) | [10] |
|  |  | 97.5% = Logistic (0.9055, 81.8654, -20.6504) | [10] |
| bite_prob_sameHH | probability of bite given a contact within the same household | Unif(0.01,0.05) | assumption, high chance of bite assumed as dogs from same household are living close together |
| increasedBiteProb_sameHH | increase of the bite probability when the dog is becoming rabid | 3 | assumption, higher chance of bite assumed for dogs showing clinical signs |
| bite_prob_betweenHH | probability of bite given a contact between dogs from different households | Unif(0.05,0.1) | assumption, less than within household contacts |
| increasedBiteProb_betwHH | increase of the bite probability when the dog is becoming rabid | 3 | assumption, higher chance of bite assumed for dogs showing clinical signs |
| transmissionProb | probability of rabies transmission given a bite (regardless of type of contact) | Pert(0.45,0.49,0.52) | [4] |
| between district movement parameters | | | |
| movements_permanent | daily frequency of permanent movements per dog, can be defined for each district separately | Unif (0.000146,0.000146) for all districts | [1] |
| movements_shortTerm | daily frequency of short term movements per dog, can be defined for each district separately | 0 | [1] |
| probs | matrix defining the relative probability of between district dog movements from one district to another in the same region | 4 to neighbouring districts; 1 to other district | [1] |
| Rabies detection parameters | | | |
| detectPeriod_firstCase | time (days) for detection of rabies after the start of clinical signs for the first case | Pert(14,21,28) | assumption; relatively short compared to [11] due to increased disease awareness |
| detectPeriod_secondCases | time (days) for detection of rabies after the start of clinical signs for all other cases in the region | Pert(1,2,4) | assumption |
| control strategy vaccination | | | |
| reactVacc_covLevel_reference | definition of the vaccination coverage can be based on the dog population (i.e. a given percentage of dogs will be vaccinated irrespective of whether they are living in the same or different household; "dog") or on the household level (i.e. a given percentage of households will be vaccinated and all dogs in that household are vaccinated; "household") | “dog” | - |
| vaccination_goal | vaccination goal is either "community" or "region", indicating whether the goal of the vaccination strategy is to vaccinate all dogs in the community or region where rabies is detected | “region” | - |
| start_vacc_delay | time (days) between the detection of the first rabid dog and the start of the vaccination campaign | 7 | assumption |
| vacc_capacity | maximum number of dogs vaccinated per day | 50 | assumption |
| reactive_vacc_cov | immunization coverage of the three roaming categories | block design of 0.5, 0.7 and 0.9 | - |
| protectionDelay | delay (days) between vaccination of the dog and the protection of the dog via vaccination. There is no protection from vaccination prior to when protection is reached | Unif(7,14) | [12-14] |
| vaccEfficacy | efficacy of the vaccination if the dog is vaccinated before last_vacc day after the exposure of the dog to rabies | Unif(0.92,0.96) | [12,13,15,16] |
| reduced_vaccEfficacy | efficacy of the vaccination if the dog is vaccinated before last_vacc day after the exposure of the dog to rabies | Unif(0.05,0.25) | assumption |
| late_vacc | number of days determines the change of the vaccination efficacy (from vaccEfficacy to reduced_vaccEfficacy) | Unif(2,4) | [17,18] |

**References**

1. Dürr S, Ward MP. Development of a novel rabies simulation model for application in a non-endemic environment. PLoS Negl Trop Dis. 2015;9: 1–22. doi:10.1371/journal.pntd.0003876

2. Hudson EG, Brookes VJ, Ward MP. Demographic studies of owned dogs in the Northern Peninsula Area, Australia to inform population and disease management strategies. Aust Vet J. 2018;96(11):487-94.

3. Hudson EG, Brookes VJ, Dürr S, Ward MP. Domestic dog roaming patterns in remote northern Australian indigenous communities and implications for disease modelling. Prev Vet Med. 2017;146:52-60.

4. Hampson K, Dushoff J, Cleaveland S, Haydon DT, Kaare M, Packer C, et al. Transmission dynamics and prospects for the elimination of canine Rabies. PLoS Biol. 2009;7: 0462–0471. doi:10.1371/journal.pbio.1000053

5. Coleman PG, Dye C. Immunization coverage required to prevent outbreaks of dog rabies. Vaccine. 1996;14: 185–186.

6. Foggin CM. Rabies and Rabies-related Viruses in Zimbabwe: historical, virological and ecological aspects. University of Zimbabwe, Harare. 1988.

7. Beran GW. Rabies and Infection by Rabies-Related Viruses. In: Beran GW, editor. Handbook of Zoonoses, Section B Viral. second. Boca Raton, Florida, USA: CRC Press LLC; 1994. pp. 307–357.

8. Fekadu M, Shaddock JH. Peripheral distribution of virus in dogs inoculated with two strains of rabies virus. Am J Vet Res. 1984;45: 724–9.

9. Hudson EG, Brookes VJ, Ward MP. Assessing the Risk of a Canine Rabies Incursion in Northern Australia. Front Vet Sci. 2017;4. doi:10.3389/fvets.2017.00141

10. Hudson EG, Brookes VJ, Ward MP, Dürr S. Using roaming behaviours of dogs to estimate contact rates: the predicted effect on rabies spread. Epidemiol Infect. 2019;147:e135.

11. Townsend SE, Sumantra IP, Pudjiatmoko, Bagus GN, Brum E, Cleaveland S, et al. Designing Programs for Eliminating Canine Rabies from Islands: Bali, Indonesia as a Case Study. PLoS Negl Trop Dis. 2013;7. doi:10.1371/journal.pntd.0002372

12. Sage G, Khawplod P, Wilde H, Lobaugh C, Menachudha T, Tepsumethanon W, et al. Immune response to rabies vaccine in Alaskan dogs: failure to achieve a consistently protective antibody response. Trans R Soc Trop Med Hyg. 1993;87: 593–595.

13. Minke JM, Bouvet J, Cliquet F, Wasniewski M, Guiot AL, Lemaitre L, et al. Comparison of antibody responses after vaccination with two inactivated rabies vaccines. Vet Microbiol. 2009;133: 283–6. doi:10.1016/j.vetmic.2008.06.024

14. Kallel H, Diouani MF, Loukil H, Trabelsi K, Snoussi MA, Majoul S, et al. Immunogenicity and efficacy of an in-house developed cell-culture derived veterinarian rabies vaccine. Vaccine. 2006;24: 4856–62. doi:10.1016/j.vaccine.2006.03.012

15. Cliquet F, Verdier Y, Sagné L, Aubert M, Schereffer JL, Selve M, et al. Neutralising antibody titration in 25,000 sera of dogs and cats vaccinated against rabies in France, in the framework of the new regulations that offer an alternative to quarantine. Rev Sci Tech. 2003;22: 857–66.

16. Sihvonen L, Kulonen K, Neuvonen E, Pekkanen K. Rabies antibodies in vaccinated dogs. Acta Vet Scand. 1995;36: 87–91.

17. Clark KA, Wilson PJ. Postexposure rabies prophylaxis and preexposure rabies vaccination failure in domestic animals. J Am Vet Med Assoc. 1996;208: 1827–30.

18. Haupt W. Rabies--risk of exposure and current trends in prevention of human cases. Vaccine. 1999;17: 1742–9.
